# Square Kilometre Array Telescope—Precision Reference Frequency Synchronisation via 1f-2f Dissemination

**DOI:** 10.1038/srep13851

**Published:** 2015-09-09

**Authors:** B. Wang, X. Zhu, C. Gao, Y. Bai, J. W. Dong, L. J. Wang

**Affiliations:** 1 Joint Institute for Measurement Science, Tsinghua University, Beijing 100084, China; 2 State Key Laboratory of Precision Measurement Technology and Instruments, Department of Precision Instruments, Tsinghua University, Beijing 100084, China; 3 Department of Physics, Tsinghua University, Beijing 100084, China; 4 National Institute of Metrology, Beijing 100013, China

## Abstract

The Square Kilometre Array (SKA) project is an international effort to build the world’s largest radio telescope, with a one-square-kilometre collecting area. In addition to its ambitious scientific objectives, such as probing cosmic dawn and the cradle of life, the SKA demands several revolutionary technological breakthroughs, such as ultra-high precision synchronisation of the frequency references for thousands of antennas. In this report, with the purpose of application to the SKA, we demonstrate a frequency reference dissemination and synchronisation scheme in which the phase-noise compensation function is applied at the client site. Hence, one central hub can be linked to a large number of client sites, thus forming a star-shaped topology. As a performance test, a 100-MHz reference frequency signal from a hydrogen maser (H-maser) clock is disseminated and recovered at two remote sites. The phase-noise characteristics of the recovered reference frequency signal coincide with those of the H-maser source and satisfy the SKA requirements.

Benefiting from innovations in modern atomic clocks, time and frequency have become the physical quantities that can be most accurately and stably measured and controlled^1^,^2^,^3^,^4^. The powerful tools for precision time and frequency dissemination and synchronisation that have been developed^5^,^6^,^7^,^8^,^9^,^10^,^11^,^12^, although initially intended for metrology^13^, can be utilised in an increasing number of fields by converting measurements of target parameters into measurements of time and frequency. For example, in radio telescope array applications^14^,^15^,^16^, the image fidelity is related to the accuracy and stability of the phase measurements. High-precision, short-term (1–100 s) phase (frequency) synchronisation among different telescope dishes is required. This requirement typically cannot be satisfied by traditional synchronisation methods via satellite links, which can achieve only long-term (>1000 s) frequency synchronisation^17^,^18^. Furthermore, satellite-based time and frequency synchronisation cannot meet the radio silence requirement of radio astronomy observations. In this regard, recently developed fibre-based frequency synchronisation methods are more suitable because they can achieve ultra-high-precision phase (frequency) synchronisation for integration times from 1 to 10^6^ s 19. To increase the availability and accessibility of this approach, several fibre-based multiple-access frequency dissemination schemes have been proposed and demonstrated^20^,^21^,^22^,^23^,^24^,^25^. However, there are still many unsolved problems related to these methods.

There are three major requirements for fibre-based reference frequency synchronisation systems that are specific to the SKA^14^. First, in the first phase of the SKA (SKA1), the synchronisation system must distribute the reference frequency signal to hundreds of receivers in a star-shaped topology. This requirement will be extended to thousands of receivers in the second phase (SKA2). Second, in accordance with the coherence requirements for the reference frequency signals, which must support astronomical observations at frequencies of up to 20 GHz with a maximum coherence loss of 2% over periods of 1–100 s, the frequency dissemination stability should be better than 1 × 10^−12^/s, 1 × 10^−13^/10 s and 1 × 10^−14^/100 s, respectively. Finally, in accordance with the jitter requirements for the recovered sampling clock at each receptor, the phase noise of the reference frequency signal should not degrade through dissemination. Simply speaking, the frequency stability and phase-noise specifications of the H-maser clock at the SKA central station should be recovered at each dish site. These requirements cannot be well satisfied by current fibre-based frequency dissemination schemes with active compensation. In all present schemes, the phase-noise detection and compensation functions are typically applied at the transmitting site. In the case of SKA1, for which hundreds of telescope dishes are planned, a corresponding number of compensation modules would need to be placed at the same central station. This requirement would incur extraordinary space requirements and cause unnecessary complexities and difficulties for future expansion. Furthermore, the phase-noise compensation bandwidth is related to the propagation delay of light in the fibre, which would cause so-called delay-unsuppressed fibre noise.

In this report, we propose and demonstrate a new frequency dissemination and synchronisation method that features phase-noise compensation performed at the client (dish) site. One central transmitting module can thus be linked to multiple client sites, and future expansion to additional receiving sites will not disrupt the structure of the central transmitting station. As a performance test, using two 50-km fibre spools, we recover 100-MHz disseminated reference frequency signals at two separate remote sites. Relative frequency stabilities between the two recovered frequency signals of 3.7 × 10^−14^/s and 3.0 × 10^−17^/10^5^ s are obtained; these values far exceed the SKA requirements. Furthermore, the phase noise of the recovered 100-MHz signal is measured and found to coincide with that of the H-maser clock’s source signal at low frequency offsets (<60 Hz) and to be better at higher frequency offsets. The proposed scheme can well satisfy all frequency dissemination and synchronisation requirements of the SKA.

## Results

### Client-side, 1f-2f actively compensated frequency dissemination method

**Figure 1:**
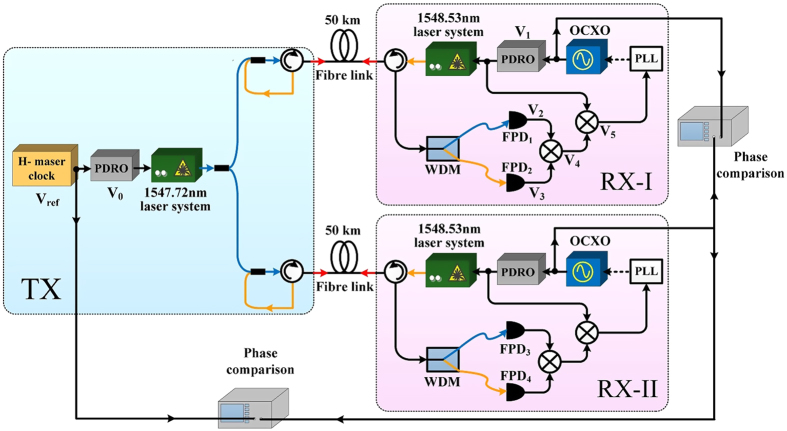
Schematic diagram of the client-side, 1f-2f actively compensated frequency dissemination system. PDRO: phase-locked dielectric resonant oscillator. OCXO: oven-controlled crystal oscillator. WDM: wavelength-division multiplexer. FPD: fast photodiode.

Figure 1 presents a schematic diagram of the client-side actively compensated frequency dissemination system. One transmitting site (TX), which represents the central station, is connected to two receiving sites (RX). As a performance test and for convenience of phase-difference measurements, TX is linked to RX-I and RX-II via two 50-km fibre spools. In our case, unlike in current active phase compensation schemes, the functions of TX are very simple—modulating and broadcasting. To increase the signal-to-noise ratio for compensation, the 100-MHz reference frequency (V_ref_ from an H-maser clock) is boosted to 2 GHz via a phase-locked dielectric resonant oscillator (PDRO); this signal can be expressed as *V*_0_ = cos(*ω*_0_t + *ϕ*_0_) (without considering its amplitude). V_0_ is used to modulate the amplitude of a 1547.72-nm diode laser. As an example, in the dissemination channel from TX to RX-I, after passing through a “2 to 1” fibre coupler and an optical circulator, the modulated laser light is coupled to the 50-km fibre link. The structure of the RX site is relatively complex. A 1-GHz PDRO, which can be expressed as *V*_1_ = cos(*ω*_1_t + *ϕ*_1_), is phase locked to a 100-MHz oven-controlled crystal oscillator (OCXO). The phase of the OCXO can be controlled by applying an external voltage. V_1_ is used to modulate the amplitude of a 1548.53-nm diode laser. With the aid of two optical circulators at the RX-I and TX sites, the modulated 1548.53-nm laser light propagates through the same 50-km fibre spool via the route from RX-I to TX and back. After the round-trip propagation, the 1548.53-nm laser light can be separated from the received one-way 1547.72-nm laser light by a wavelength-division multiplexer (WDM). The modulated 1547.72-nm laser is then detected by a fast photodiode (FPD1). The directly received 2-GHz frequency signal from TX can be expressed as *V*_2_ = cos(*ω*_0_t + *ϕ*_0_ + *ϕ*_p_), where *ϕ*_*p*_ is the phase fluctuation induced during the 50-km fibre dissemination. The modulated 1548.53-nm laser carrier is detected by FPD2, and the recovered 1-GHz frequency signal can be expressed as 
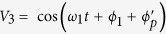
, where 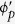 is the phase fluctuation induced by the 100-km (round-trip) fibre dissemination. We then down mix the signals V_2_ and V_3_ to obtain 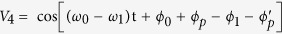. Furthermore, down-mixing the signals V_1_ and V_4_ yields an error signal 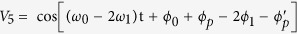. Finally, V_5_ is used to close the phase-locked loop (PLL) to control the phase of the OCXO. In this case, the relation *ω*_0_ = 2*ω*_1_ is satisfied, and under a fibre dispersion condition (discussed later), the one-way phase fluctuation of the 2-GHz frequency signal is the same as the round-trip accumulated phase fluctuation of the 1-GHz frequency signal, 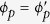. Consequently, the error signal can be expressed as *V*_5_ = cos(*ϕ*_0_ − 2*ϕ*_1_). When the PLL is closed, the phase of the OCXO at RX-I is locked to the phase of V_ref_ at TX, with *ϕ*_0_ = 2*ϕ*_1_, thus providing stable-frequency dissemination from TX to RX-I. As previously mentioned, the light propagation delay in the fibre link limits the feedback control time and cause delay-unsuppressed fibre noise. To ensure that the phase noise of the 100-MHz OCXO at the client site is not worsened by this unsuppressed fibre noise, we use a custom-designed narrow-bandwidth PLL. Its bandwidth can be tuned and set based on the phase-noise characteristics of the free-running OCXO and H-maser clock. In our case, the PLL’s bandwidth is set to 10 Hz. The same structure can be applied to RX-II and all other future sites. The method is referred to as the “1f-2f” method because of the factor-of-2 relationship between the TX and RX frequencies.

### Measurements of the frequency dissemination stability

**Figure 2:**
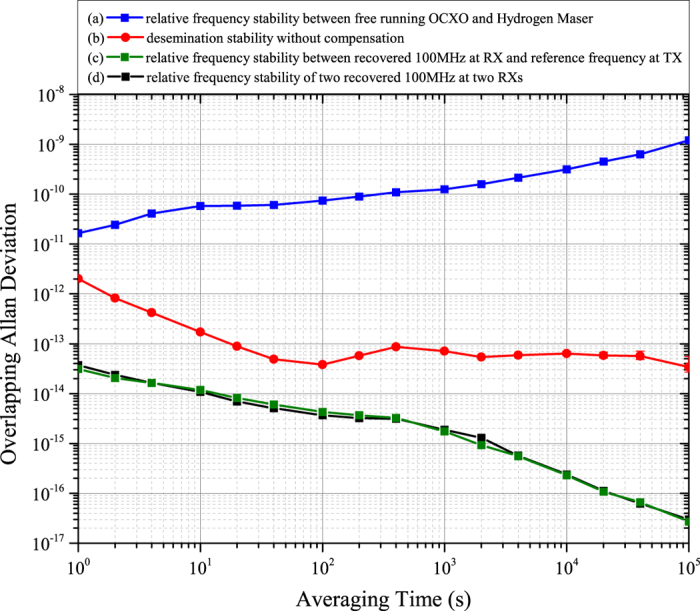
Results of relative frequency stability measurements. (**a**) Relative frequency stability between the free-running OCXO and H-maser clock. (**b**) Measured frequency stability of the dissemination system without compensation. (**c**) Relative frequency stability between the recovered 100-MHz signal at RX and V_ref_ at TX with the PLL closed. (**d**) Relative frequency stability of the two recovered 100-MHz signals at the two RX sites with both PLLs closed. Curves (**c**,**d**) were measured simultaneously.

To test the dissemination stability of the proposed client-site active compensation method, we performed a series of measurements. The results are shown in Fig. 2. Curve (a) represents the measured relative stability of the 100-MHz frequency signals between the H-maser at TX and the OCXO at RX-I with the PLL open. The relative frequency stabilities of 1.6 × 10^−11^/s and 1.2 × 10^−9^/10^5^ s reflect the frequency stability of the OCXO that we used. Curve (b) represents the relative frequency stability between *V*_*0*_ and *V*_*2*_. The relative frequency stabilities of 2.0 × 10^−12^/s and 3.4 × 10^−14^/10^5^ s reflect the dissemination stability of the 50-km fibre without compensation. Curve (c) represents the measured relative stability of the 100-MHz frequency signals between the H-maser clock at TX and the OCXO at RX-I with the PLL closed at a 10-Hz effective bandwidth. Relative frequency stabilities of 3.1 × 10^−14^/s and 2.7 × 10^−17^/10^5^ s were obtained, thus indicating that the OCXO at RX-I had become phase locked to the H-maser at TX. We also measured the relative frequency stability between the two OCXOs at RX-I and RX-II when the phase-locked loops at both dissemination links were closed, as represented by curve (d). Relative frequency stabilities of 3.7 × 10^−14^/s and 3.0 × 10^−17^/10^5^ s were achieved, thereby indicating that the OCXOs at the two RX sites were phase coherent with each other within the compensation bandwidth of the dissemination system.

### Phase-noise measurements

**Figure 3:**
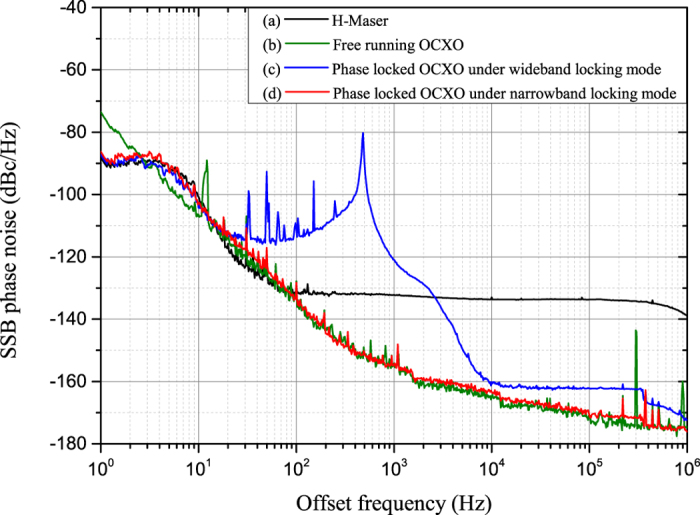
Results of phase-noise measurements for various 100-MHz frequency signals. (**a**) SSB phase noise of the H-maser at TX. (**b**) SSB phase noise of the free-running OCXO used in the experiment. (**c**) SSB phase noise of the phase-locked OCXO in wideband locking mode. (**d**) SSB phase noise of the phase-locked OCXO in narrowband locking mode.

In the above measurements, we tested the frequency dissemination stability for integration times from 1 to 10^5^ s, i.e., the long-term stability (longer than 1 s) of the recovered 100-MHz frequency signal. However, outstanding short-term stability (shorter than 1 s) for the recovered 100-MHz frequency signal is also required by the SKA. In the frequency domain, the short-term phase instability can be defined in terms of the one-sided spectral density 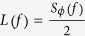, which is half of the double-sided spectral density of phase fluctuations, *S*_*ϕ*_(*f*) (including the fluctuations in both the upper and lower sidebands of the carrier)^26^. Typically, L(f) is expressed in decibels (dB) as 10*Lg*[*L*(*f*)] and is referred to as the single-sideband (SSB) phase noise. This quantity was directly measured using a commercial signal source analyser (Agilent E5052B). Figure 3 shows the SSB phase-noise spectra of the 100-MHz signals measured in various cases. Curves (a) and (b) represent the measured SSB phase-noise spectra of the H-maser clock and free-running OCXO, respectively, that we used. Obviously, the long-term phase stability of the free-running OCXO is worse than that of the H-maser clock, whereas its high-frequency-offset (>60 Hz) phase-noise characteristics are better than those of the H-maser clock. The main crossing point of these two spectra occurs at approximately 10 Hz. Curve (c) represents the SSB phase noise of the OCXO when the PLL was closed in a wideband locking mode. In this case, the loop bandwidth is determined by the light propagation delay and can be calculated as 1/4τ ~ 500 Hz^27^, where τ is the round-trip propagation time for light in the 50-km fibre link. Consequently, there is a large bump in curve (c) at a frequency offset of approximately 500 Hz. Furthermore, the unsuppressed high-frequency phase noise (>500 Hz) induced during fibre dissemination worsens the phase-locked OCXO’s phase noise. Curve (d) represents the SSB phase noise of the OCXO when the effective bandwidth of the PLL is set to 10 Hz. In this case, the SSB phase noise of the OCXO coincides with that of the H-maser at frequency offsets of less than 60 Hz and is even better at frequency offsets higher than 60 Hz. Based on these measurements, we observed that the long-term stability of the recovered 100 MHz frequency signal was locked to the H-maser at the TX site and that its short-term phase-noise characteristics can be improved by carefully choosing the locking bandwidth.

## Discussion

In the proposed scheme, the wavelengths of the two laser carriers are different. Consequently, the fibre dispersion may cause a phase-delay difference between the two laser carriers, which may limit the frequency dissemination stability. Here, we discuss this phenomenon and analyse its effects in a real-world situation. The phase time delay *τ*(*λ*, *T*) of the disseminated frequency signal is
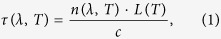
where c is the speed of light in vacuum, *n*(*λ*, *T*) is the refractive index of the fibre at optical wavelength *λ* and temperature T, and *L*(*T*) is the fibre length. In practice, temperature fluctuations in the fibre link are the dominant factor that will degrade the frequency dissemination stability. We calculate the second-order partial derivative of the phase time delay as follows:
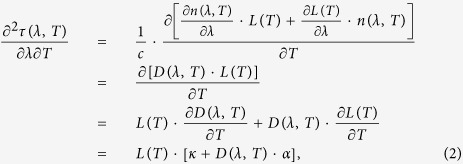
where the fibre dispersion is
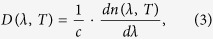
the thermal coefficient of chromatic dispersion is
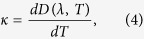
and the thermal expansion coefficient for the fibre length is
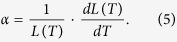
For a commercial G652 fibre, *α* = 5.6 × 10^−7^/°*C* 28, *D* = 17 *ps*/(*nm*·*km*) and *κ* = −1.45 × 10^−3^ *ps*/(*km*·*nm*·°*C*)^29^ at approximately 1550 nm. Obviously, the thermal coefficient of chromatic dispersion is the dominant factor that controls the phase time delay difference. For dissemination over a 100-km fibre, the phase time delay difference is *δτ* = −0.14 *ps*/(*nm*·°*C*). Supposing a diurnal temperature fluctuation of 30 °*C*, the difference in the phase time delays of the frequency signals carried by 1547.72- and 1548.53-nm carriers (a 0.81-nm difference) will be 3.5 ps, which corresponds to a dissemination stability of 8.1 × 10^−17^ over an integration time of half a day. This frequency dissemination stability can satisfy the requirements of almost all current practical applications. Using the dense wavelength-division multiplexing (DWDM) technique, the wavelengths of the two laser carriers can be within 0.4 nm of each other, and this will further reduce the impact of chromatic dispersion on the dissemination stability.

## Conclusions

Unlike all current fibre-based frequency dissemination schemes, in this proposed and demonstrated 1f-2f scheme, the phase-noise compensation function is applied at the receiving site. This key change makes this method suitable for use in any star-shaped topology frequency dissemination applications. The space requirements and complexity of the frequency dissemination system at the central station can be dramatically reduced. Furthermore, in this scalable scheme, future expansions in terms of the number of receiving sites will not disrupt the structure of the central transmitting station. This approach can satisfy all three basic requirements for SKA frequency reference synchronisation as mentioned earlier. Further verification on a telescope array will be performed in the near future.

## Methods

### Dissemination stability measurements

As noted above, curves (c) and (d) in Fig. 2 were measured simultaneously. However, only one commercial phase-noise measurement device (model 5125A, Microsemi Corporation) was available. Consequently, we used the “voltmeter” method to measure the dissemination stability with active compensation. During the measurement, two 100-MHz signals were mixed into a direct current (DC) signal. The voltage fluctuation of this DC signal corresponded to the relative phase fluctuation between the two 100-MHz signals. The voltage fluctuation was measured using a precise voltmeter and recorded by a computer. The relative phase time fluctuation was calculated using 
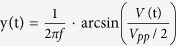
. In this formula, *f* is the frequency of the comparison signal, with the relation *ω* = 2*πf* (here, *f* = 100 MHz); *V*(*t*) is the measured real-time voltage fluctuation of the DC signal; and *V*_*pp*_ is the peak-to-peak voltage of the DC signal when the relative phase change between the two comparison signals is greater than 2*π*, which can be measured when the PLL is open. Using the commercial software package “Stable 32”, we calculate the overlapping Allan deviation from the measured phase time fluctuation. In practice, to enhance the sensitivity of phase-fluctuation measurements, *V*(*t*) is always initially set to approximately 0. This can be achieved by choosing a suitable length for the radio frequency (RF) cable between the OCXO and phase comparison mixer. Here, the result measured using the voltmeter method was verified using a model 5125A commercial device. The two results were found to be identical. Compared with the 5125A, the voltmeter method offers certain advantages. The frequency measurement range of the 5125A is 1–400 MHz, whereas the voltmeter method can be used to measure frequencies higher than 400 MHz. The disadvantages of the voltmeter method are the following: (1) it cannot be used to measure signals with different frequencies, and (2) it cannot be used to measure unstable signals whose relative phase fluctuation is greater than 2*π*, e.g., curves (a) and (b) in Fig. 2.

## Additional Information

**How to cite this article**: Wang, B. *et al.* Square Kilometre Array Telescope—Precision Reference Frequency Synchronisation via 1f-2f Dissemination. *Sci. Rep.* **5**, 13851; doi: 10.1038/srep13851 (2015).
